# Oral health in southeast Asia: addressing inaccessibility

**DOI:** 10.1016/j.lansea.2024.100528

**Published:** 2025-01-10

**Authors:** The Lancet Regional Health – Southeast Asia

Oral health, although important physically, mentally, and socially, is widely neglected in general health care. WHO defines oral health as “the state of the mouth, teeth and orofacial structures that enables individuals to perform essential functions such as eating, breathing and speaking, and encompasses psychosocial dimensions such as self-confidence, well-being and the ability to socialise and work without pain, discomfort and embarrassment”. The World Health Assembly in 2021 passed a resolution encouraging its Member States to include oral health care in their non-communicable disease agenda and universal health coverage. The estimated number of cases of oral diseases worldwide increased by more than 1 billion from 1990 to 2019. In 2019, the highest number of cases related to oral disease (903 million) was reported in the southeast Asia region (SEAR), which harbours a quarter of the world's population. However, dental care services are inadequate in SEAR, leading to productivity loss of US$13 billion. There is hence a need to address this inadequacy in line with the 2022 resolution of the WHO Regional Committee for South-East Asia for non-communicable diseases and oral health.

Oral health issues include dental problems such as dental caries (tooth breakdown), severe periodontal disease (gum disease), and edentulism (tooth loss). In 2019, the estimated prevalence of permanent teeth caries (age >5 years) was highest in Timor-Leste, Nepal, and Bangladesh in SEAR. Furthermore, estimated prevalence of severe periodontal disease (age >15 years) was highest in Bhutan, Bangladesh, and India, whereas Indonesia, Timor-Leste, and North Korea ranked the highest for edentulism (age >60 years). SEAR has an ageing population, and dental problems will likely increase in the coming years. In SEAR, dental services are mainly provided by the private sector. Countries like India and Thailand have medical tourism which also includes dental services and cosmetic dentistry. Unfortunately, as most of the dental clinics are concentrated in cities, people from rural regions and indigenous people who live in remote areas must travel to receive better treatment. Also, people with low income are less keen to prioritise oral health due to out-of-pocket expenses.

Smokeless tobacco and smoking can significantly impact dental health and increase the risk of oral cancer. SEAR has the highest tobacco use (26.5%) among the WHO regions. Although the recent trends show a decline in tobacco use worldwide, we must not be complacent. In 2022, 98 735 people died due to cancers related to the lip and oral cavity in SEAR. Furthermore, cancers related to the lip and oral cavity were the most frequent cancers among males in SEAR. Indigenous people are prone to dental health issues and oral cancer due to cultural factors associated with tobacco use. Aggressive public awareness campaigns would help reduce the use of tobacco, especially smokeless tobacco.

Dental caries can be further complicated by foods with high sugar content. Free sugars (especially those in foods with high sugar content) are metabolised by bacteria, leading to acid production and the demineralisation of tooth enamel and dentine. Sugar-sweetened beverages (SSBs) are addictive, and sometimes more accessible than water. India has the highest tax rate for SSBs in the world, and is considering another proposal for further increasing tax for SSBs and tobacco products. Will such an elevation in tax reduce the use of tobacco or SSBs? Or would it benefit the government? A simulation analysis from Thailand showed that taxes on SSBs alone would not majorly impact oral health; there would also be a need for public policies to regulate sugar content in food sources other than SSBs. There is a requirement for stricter regulations and improved front-of-pack labelling with respect to sugar content in packaged foods. Healthy behaviour could be initiated during childhood itself via the school curriculum.

Regular checkups through dental camps or mobile units in rural areas and schools would help address the inequities associated with accessibility. There must be increased budget allocation, improved oral health workforce planning, and collaboration between the private and public sectors to reach marginalised communities and older people in rural areas. During the COVID-19 pandemic, dental services were affected due to the lockdown period in all countries, with many patients opting for teledentistry. Although there was an increased risk of viral transmission during routine oral checks, dentists adapted efficiently by introducing infection control strategies such as sterilisation and ventilation. Interestingly, in Thailand, dental assistants had an elevated risk for SARS-CoV-2 infection due to inadequate personal protective equipment. The health authorities must be mindful of this challenge if another pandemic arises.

*The Lancet Regional Health – Southeast Asia* invites original research articles, systematic reviews, meta-analyses, health policies, viewpoints, personal views, comments, and commentaries on improving oral health in SEAR. A previous Comment by Reddy and Colleagues called for additional research on dental surgical techniques in India. Research on new interventions and cheaper technologies would improve oral health care and address inequities. Access to oral health care should not be a privilege but a universal right.

